# Peer review of "A Narrative Review of the Efficacy of Interventions for Emotional Dysregulation, and Underlying Bio–Psycho–Social Factors"

_brainsci, 2024, doi:10.3390/brainsci14050453_

Round 1

Reviewer 1 Report

Comments and Suggestions for Authors

GENERAL COMMENTS

This is an interesting and complete narrative review on interventions targeting emotion dysregulation.  The topic holds relevance within the ongoing discussion about the pressing necessity for early interventions targeted at transdiagnostic features across common mental health disorders. The viewpoints presented by the authors are overall well-supported and clinically pertinent.

I have just a few corrections that are needed to highlight the clinical relevance of the research question investigated and to improve the clarity of the work and of the terms employed.

ABSTRACT

The abstract effectively conveys the message of the review.

Please mention limitations in the abstract.

INTRODUCTION

The Background is overall appropriate but a critical and more in-depth discussion of why it is crucial to focus specifically on emotion regulation is critically needed, especially from biological viewpoint. Social and psychological factors are overall appropriate, but, for instance, the authors may briefly mention that emotion dysregulation, besides being a transdiagnostic symptom and risk factor for psychiatric disorders, has also been associated to somatic problems, such as a chronic pro-inflammatory status (e.g. see a recent review on the topic: “Inflammation and emotion regulation: a narrative review of evidence and mechanisms in emotion dysregulation disorders”, DOI: 10.1042/NS20220077). This is also relevant concerning the HPA axis, stress, and the vagus nerve that the authors mention in the following section.

Please briefly describe your definitions of “irritability” and “transdiagnostic” in the background.

METHODS

The methods are sound, for a narrative review.

MAIN BODY

The discussion overall provides interesting information.

Emotion regulating circuits may maturate up until 25 years of age, please briefly discuss relevance and differences of the results between age groups.

LIMITATIONS

-       Please include a subsection dedicated to limitations (of the existing literature and of the review itself)

Author Response

Comment 1: This is an interesting and complete narrative review on interventions targeting emotion dysregulation.  The topic holds relevance within the ongoing discussion about the pressing necessity for early interventions targeted at transdiagnostic features across common mental health disorders. The viewpoints presented by the authors are overall well-supported and clinically pertinent.

The abstract effectively conveys the message of the review. The Background is overall appropriate. (..). The methods are sound, for a narrative review. The discussion overall provides interesting information.

I have just a few corrections that are needed to highlight the clinical relevance of the research question investigated and to improve the clarity of the work and of the terms employed.

Response: We thank the Reviewer for their positive feedback.

Comment 2: Please mention limitations in the abstract.

Response: We have now added a brief statement in the abstract, highlighting the limitations of our work, see page 1:

“Although our review was not intended as a traditional systematic review, and the systematic search was only restricted to systematic reviews and meta-analyses, (..)”

Comment 3: (..) a critical and more in-depth discussion of why it is crucial to focus specifically on emotion regulation is critically needed, especially from biological viewpoint. Social and psychological factors are overall appropriate, but, for instance, the authors may briefly mention that emotion dysregulation, besides being a transdiagnostic symptom and risk factor for psychiatric disorders, has also been associated to somatic problems, such as a chronic pro-inflammatory status (e.g. see a recent review on the topic: “Inflammation and emotion regulation: a narrative review of evidence and mechanisms in emotion dysregulation disorders”, DOI: 10.1042/NS20220077). This is also relevant concerning the HPA axis, stress, and the vagus nerve that the authors mention in the following section.

Response: Thanks for sharing the reference, which is very interesting. We have added, on page 3, the following sentence:

“Associations between ED and chronic inflammation have also been found [35], suggesting that chronic inflammation since early life could be a key factor impacting the development of brain structures involved in ER and, therefore, contributing to the onset and maintenance of ED and internalizing/externalizing symptoms.”

Comment 4: Please briefly describe your definitions of “irritability” and “transdiagnostic” in the background.

Response: When introducing “irritability” and “transdiagnostic”, we have now provided brief definitions, as follows:

“irritability (namely, increased proneness to experience and/or express negative emotional states)”

“transdiagnostic (namely, present across different disorders)”

Comment 5: Emotion regulating circuits may maturate up until 25 years of age, please briefly discuss relevance and differences of the results between age groups.

Response: We have added the following sentences, on page 3 and 14, respectively, as follows:

“The optimal development of the PFC (which is not fully mature until young adulthood) is particularly relevant as it supports several processes linked to ER, such as effortful control [25], executive functions [26], and goal-directed behavior [27]. Moreover, as structural and functional connectivity between pre-frontal, limbic and brainstem systems gradually increase throughout development, basic extrinsic ER strategies (such as calming down, after experiencing stress, with a pacifier or when hugged by parents) evolve into more elaborated and intrinsic strategies at a later age [28].”

“The fact that ER circuits continue to mature until approximately the age of 25, highlights the importance of focusing on understanding developmental trajectories of ER skills maturation. It could also explain why differences in emotional processing and regulation between younger adolescents and older adults are often evident.”

Comment 6: Please include a subsection dedicated to limitations (of the existing literature and of the review itself).

Response: We have now added the paragraph “6. Clinical implications and Future directions”, on page 14, where we discuss some limitations of our work, and gaps in the reviewed literature that shall be addressed in future research.

Reviewer 2 Report

Comments and Suggestions for Authors

A narrative review of the efficacy of interventions for emotional dysregulation and underlying bio-psycho-social factors

I read the manuscript with interest and the authors can find my appraisal (section-by-section), concerns, and recommendations as follows:

The introduction is well-written and informative. However, I found the statement in line 40 “A categorization of ER strategies as adaptive or maladaptive is often portrayed 40 in the literature” less clear and I invite the authors to clarify it in the section, or, at least, expand it better. Moreover, at the end of the section, the authors disclosed that a previous meta-analysis has been carried out and reported the items of the systematic search performed in PubMed. This is correct, but at the end of the section, the aims or a brief introduction to the content of the narrative review is preferable. I know, based on my experience,  that this is difficult, as well as writing a narrative review, but it helps to improve the quality of the manuscript. Similarly,  I advise putting the methodological section (the systematic search) at the end of the introduction in a specific subsection following the introduction.

There is no section 2. This needs to be fixed.

Section 3. The section is also informative in terms of the content and I have appreciated the schema that the authors chose. However, the term effortful control needs to be revised. The authors wrote about the development of PFC and its association with executive functions, in which inhibitory control plays a key role. Indeed, inhibitory control and development need to be treated with care. The authors are invited to add specific trajectories in the development of executive functions, taking into account that inhibition which is an important step in neurocognitive development , has a role in adolescence and in the transition between adolescence and adulthood.

Similarly, the authors introduced the HPA in a good way. However, HPA and HPG are two related axes that are in interplay for different functions, including stress. This needs to be briefly discussed and added to the paragraph.

In the paragraph about the ANS, the authors delineated the relationship between ER and autonomic changes including HRV. However, more info is needed about breath and galvanic response. These are important indicators of stress and emotional reactivity in several situations.

3.2. Childhood adverse experiences and psycho-social factors- I found it very complete. However, the authors in the previous paragraph added more information about neurocognitive factors. Here, according to me, they need to follow a similar line, adding more about the connection between adverse experiences and neuro-psycho-social factors. However, adverse experiences are not only related to external factors but also to more “proximal” factors (i.e. adverse experiences related to an illness during childhood).

4.1. Pharmacological interventions in adults. Interesting and well-written. I recommend adding a summary, brief, and critical, at the end of the section. The same for the section “4.2. Pharmacological interventions in children and adolescents”. I was expecting a critical view of this.

I found very interesting the section about psychotherapies. According to me, the info included is correct and explicative. However, reading the section, I asked “And what about brain changes?” About CBT several imaging studies assessed the re-organization of the brain in different types of patients after a CBT training/ therapy. Here, this information is lacking and, according to me, this is a very important part for the readers of this journal. This advice is for all the subsections until 4.2.8 included.

4.2.10 Food Supplements- according to me, the information about Omega 3 needs to be taken into account carefully. In which manner the Omega 3 can be helpful to ER? Here also animal evidence could be added.

 The Conclusion is well written. 

Author Response

Comment 1: I read the manuscript with interest.

The introduction is well-written and informative.

Section 3 (..) is also informative in terms of the content and I have appreciated the schema that the authors chose.

Similarly, the authors introduced the HPA in a good way. In the paragraph about the ANS, the authors delineated the relationship between ER and autonomic changes including HRV.

3.2. Childhood adverse experiences and psycho-social factors- I found it very complete.

4.1. Pharmacological interventions in adults. Interesting and well-written

I found very interesting the section about psychotherapies. According to me, the info included is correct and explicative.

The Conclusion is well written.

Response: We thank the Reviewer for their positive feedback.

Comment 2: I found the statement in line 40 “A categorization of ER strategies as adaptive or maladaptive is often portrayed 40 in the literature” less clear and I invite the authors to clarify it in the section, or, at least, expand it better.

Response: We have now specified, on page 1, that:

“A categorization of ER strategies as adaptive or maladaptive is often portrayed in the literature [5]. For example, cognitive reappraisal (which involves changing the cognitive meaning of a situation or event, and its emotional valence), emotional acceptance, and attempting to change the nature of a negative situation by eliminating the source of stress (problem solving), are associated with better interpersonal functioning, greater wellbeing, and positive affect. Conversely, emotional avoidance (ignoring the source of stress) and suppression of facial expressions and emotional behaviors, have been found to lead to increased depression and anxiety, negative mood, lower social functioning and reduced psychological well-being [6].”

Comment 3: Moreover, at the end of the section, the authors disclosed that a previous meta-analysis has been carried out and reported the items of the systematic search performed in PubMed. This is correct, but at the end of the section, the aims or a brief introduction to the content of the narrative review is preferable. I know, based on my experience, that this is difficult, as well as writing a narrative review, but it helps to improve the quality of the manuscript.

Response: We have now moved the description of the methodological component of our review, in section “2. Search strategy”. Here, we have specified how we retrieved previously published systematic reviews and meta-analysis (not conducted by the current research team), which we then reviewed in sections 3-5. In relation to the Reviewer’s comment, we have now specified, on page 2:

“The retrieved articles were screened by TEC, and cross-checked by AB, to identify available research on factors underlying ER and ED (reported in section 3), and evidence of the effectiveness of different pharmacological (section 4) and non-pharmacological interventions (section 6) for reducing ED or promoting ER.”

Comment 4: I advise putting the methodological section (the systematic search) at the end of the introduction in a specific subsection following the introduction. (..) There is no section 2. This needs to be fixed.

Response: Thanks for pointing this out. We had accidentally deleted the heading for “Search strategy”, which has now been added to the manuscript (section 2).

Comment 5: Section 3. the term effortful control needs to be revised. The authors wrote about the development of PFC and its association with executive functions, in which inhibitory control plays a key role. Indeed, inhibitory control and development need to be treated with care. The authors are invited to add specific trajectories in the development of executive functions, taking into account that inhibition which is an important step in neurocognitive development, has a role in adolescence and in the transition between adolescence and adulthood.

Response: Based on our review of the literature, both effortful control and response inhibition are indeed discussed in relation to emotion regulation. To address the Reviewer’s comment, we have added the following sentences to the text, on page 3:

“The optimal development of the PFC (which is not fully mature until young adulthood) is particularly relevant as it supports several processes linked to ER, such as effortful control [26], executive functions [27], and goal-directed behavior [28]. Moreover, as structural and functional connectivity between pre-frontal, limbic and brainstem systems gradually increase throughout development, basic extrinsic ER strategies (such as calming down, after experiencing stress, with a pacifier or when hugged by parents) evolve into more elaborated and intrinsic strategies at a later age [29].”

Comment 6: HPA and HPG are two related axes that are in interplay for different functions, including stress. This needs to be briefly discussed and added to the paragraph.

Response: Thanks for pointing out this aspect; we have added a reference and mentioned the HPG together with the HPA, as follows, on page 3:

“Another neurobiological system possibly involved in ER and ED is the hypothalamic–pituitary–adrenal (HPA) axis, a group of endocrine structures which – together with the hypothalamic-pituitary-gonadal (HPG) reproductive axis [34]”

Comment 7: However, more info is needed about breath and galvanic response. These are important indicators of stress and emotional reactivity in several situations.

Response: We have now added the following sentence, on page 4:

“When experiencing stress and during situations that require fast allocation and mobilization of energetic resources, the activation of the sympathetic branch of the ANS produces excitatory effects on bodily systems, such as increases in HR, galvanic skin responses, faster respiration rate, and pupil dilations.”

Comment 8: 3.2. Childhood adverse experiences and psycho-social factors- the authors in the previous paragraph added more information about neurocognitive factors. Here, according to me, they need to follow a similar line, adding more about the connection between adverse experiences and neuro-psycho-social factors. However, adverse experiences are not only related to external factors but also to more “proximal” factors (i.e. adverse experiences related to an illness during childhood).

Response: We would prefer to keep the two sections separate, namely discuss neuro-cognitive factors and psycho-social factors in section 3.1 and 3.2, respectively. However, we agree in relation to the inter-relation between the two, and we have added, in paragraph 3.1., a sentence suggesting what the Reviewer mentioned in their comment, namely that:

“Genetic and environmental factors (including childhood adverse experiences [23], as discussed below, and illness) may interplay and alter trajectories of brain development and/or functioning, also influencing the acquisition of ER skills across development [24].”

Comment 9: 4.1. Pharmacological interventions in adults. I recommend adding a summary, brief, and critical, at the end of the section. The same for the section “4.2. Pharmacological interventions in children and adolescents”. I was expecting a critical view of this.

Response: For both sections, we have added a summary at the beginning of each paragraph, as follows:

“Medication is often used as a treatment for mental disorders. As illustrated in more details below, there is evidence that medications commonly used to treat internalizing and externalizing symptoms (e.g., depression, ADHD, and psychotic symptoms) have beneficial effects for reducing ED and promoting ER. Pharmacological options that can have a beneficial effect on ED include antidepressants, antipsychotics, and ADHD medication.”.

“Recent evidence synthesis studies (see, for example, [60]) have shown the benefits of implementing non-pharmacological interventions, both in combination with medication and as standalone treatment, with people experiencing ED. As summarised in more details below, the most used and effective non-pharmacological interventions for ED (especially, for adults) are cognitive behavioural therapy (CBT), dialectical behavioural therapy (DBT), and mindfulness-based Therapy (MBT). There is also evidence of benefits associated with the adaptation of these traditional protocols (see the Unified Protocol for Emotional Disorder), the delivery of interventions in group settings (which promote interpersonal validation), the integration of biofeedback components (e.g., HRV-biofeedback), and the delivery of the interventions via digital technologies (e.g., mobile or virtual reality).

We have also critically appraised the evidence in section “6. Clinical implications and Future directions”.

“From a clinical standpoint, the evidence is yet insufficient to provide clear guidelines for clinicians and mental health practitioners. Studies assessing the specific effects of each intervention on the bio-psycho-social factors that have been proposed to contribute to the onset and maintenance of ED and difficulties in ER are needed to inform clinical practice. Unfortunately, our review did not identify any studies – specifically focused on ED or ER – addressing this. However, there is preliminary evidence of changes in the function and activation of fronto-limbic systems involved in emotion processing and/or regulation, with CBT and antidepressants in people with depression [87,88], CBT and SSRIs in people with anxiety [89], stimulants and non-stimulants in people with ADHD [90], DBT in people with BPD [91], and mindfulness-based interventions [92,93]. In relation to HRV, it has been shown that HRV-biofeedback produces increases in HRV, and this is reflected in reduced ED and better ER at the behavioral level [79,80]. A major limitation of the current literature is that methodological differences across studies (e.g., type of intervention and duration, active vs. passive control arm, sample characteristics, type of imaging technique used) often lead to inconsistent patterns and findings that are rarely replicated in larger follow-up studies. Adopting transdiagnostic and computational approaches to the study of the effects of different interventions for ED on brain and body functioning, could help predict what intervention works best for people with specific clinical and demographic profiles.”

Comment 10: Section about psychotherapies. I asked, “And what about brain changes?” About CBT several imaging studies assessed the re-organization of the brain in different types of patients after a CBT training/ therapy. Here, this information is lacking and, according to me, this is a very important part for the readers of this journal. This advice is for all the subsections until 4.2.8 included.

Response: Although we agree with the Reviewer’s point, we think that integrating such evidence for each of the sections 4 and 5 would distract the reader from the key messages we would like to deliver via the review. We think, however, that discussing the effects of both medication and non-pharmacological interventions on key neural systems possibly involved in ER/ED, is important. We therefore added the following paragraph to the main discussion, on page 14, as follows:

“Studies assessing the specific effects of each intervention on the bio-psycho-social factors that have been proposed to contribute to the onset and maintenance of ED and difficulties in ER are needed to inform clinical practice. Unfortunately, our review did not identify any studies – specifically focused on ED or ER – addressing this. However, there is preliminary evidence of changes in the function and activation of fronto-limbic systems involved in emotion processing and/or regulation, with CBT and antidepressants in people with depression [87,88], CBT and SSRIs in people with anxiety [89], stimulants and non-stimulants in people with ADHD [90], DBT in people with BPD [91], and mindfulness-based interventions [92,93]. In relation to HRV, it has been shown that HRV-biofeedback produces increases in HRV, and this is reflected in reduced ED and better ER at the behavioral level [79,80]. A major limitation of the current literature is that methodological differences across studies (e.g., type of intervention and duration, active vs. passive control arm, sample characteristics, type of imaging technique used) often lead to inconsistent patterns and findings that are rarely replicated in larger follow-up studies. Adopting transdiagnostic and computational approaches to the study of the effects of different interventions for ED on brain and body functioning, could help predict what intervention works best for people with specific clinical and demographic profiles.”

Comment 11: 4.2.10 Food Supplements- according to me, the information about Omega 3 needs to be taken into account carefully. In which manner the Omega 3 can be helpful to ER? Here also animal evidence could be added.

Response: We agree that we need to be cautious in relation to food supplement. In fact, we have reiterated, on page, 11, that “evidence is scarce and further research is needed before making solid conclusions”. In relation to potential mechanisms underlying beneficial effects of Omega 3 on ED, we pointed out, on page 10 that: “Considering that deficiencies in omega-3 fatty acids have been associated with violent behavior and emotional lability (related to consequent alterations in serotonin and dopamine levels) [80], omega-3 supplementation might eventually prove helpful for reducing ED (..)”. Considering the focus of the review is on humans, we did not deem helpful for the reader to add further literature on animal studies.

Reviewer 3 Report

Comments and Suggestions for Authors

The manuscript reports a narrative review of the existing literature on the efficacy of interventions for emotional dysregulation. The authors covered the spectrum of ages and types of interventions. The paper is well-written and I do not have specific concerns. I have only a few comments for the authors to improve the paper:

- paragraph 2 is missing

- there is growing evidence that child adversities might have an effect that modifies neurobiological elements. In your paper, they seem to be separate events. Please modify.

-  I think paragraph 4 should be split into different chapters because it contains different elements.

- From a clinical perspective, I might look for specific insight regarding the best approach to apply. Is it possible to discuss this aspect before the conclusion?

- have you evaluated the inclusion of a table to summarize the evidence?

- I agree that emotional dysregulation is a transdiagnostic elements, but I think that treatments reported in the review have been related to specific diagnoses. Have you evaluated if this aspect is balanced or not?

- please include a paragraph about the limits of this revision. You should be aware of them, especially the possible biases and the possible exclusion of some papers.

Author Response

Comment 1: The manuscript reports a narrative review of the existing literature on the efficacy of interventions for emotional dysregulation. The authors covered the spectrum of ages and types of interventions. The paper is well-written and I do not have specific concerns.

Response: We thank the Reviewer for their positive feedback.

Comment 2: paragraph 2 is missing

Response: Thanks for pointing this out. We had accidentally deleted the heading for “Search strategy”, which has now been added to the manuscript (section 2).

Comment 3: there is growing evidence that child adversities might have an effect that modifies neurobiological elements. In your paper, they seem to be separate events. Please modify.

Response: We have now added a sentence (to also address another comment from Reviewer #2), specifying that:

“Genetic and environmental factors (including childhood adverse experiences [23], as discussed below, and illness) may interplay and alter trajectories of brain development and/or functioning, also influencing the acquisition of ER skills across development [23]”

Comment 4: I think paragraph 4 should be split into different chapters because it contains different elements.

Response: We thank the Reviewer for this suggestion. We have now split this section in “4. Pharmacological interventions” and “5. Non-pharmacological interventions”.

Comment 5: From a clinical perspective, I might look for specific insight regarding the best approach to apply. Is it possible to discuss this aspect before the conclusion?

Response: We have critically appraised the evidence in section “6. Clinical implications and Future directions”:

“From a clinical standpoint, the evidence is yet insufficient to provide clear guidelines for clinicians and mental health practitioners. Studies assessing the specific effects of each intervention on the bio-psycho-social factors that have been proposed to contribute to the onset and maintenance of ED and difficulties in ER are needed to inform clinical practice. Unfortunately, our review did not identify any studies – specifically focused on ED or ER – addressing this. However, there is preliminary evidence of changes in the function and activation of fronto-limbic systems involved in emotion processing and/or regulation, with CBT and antidepressants in people with depression [87,88], CBT and SSRIs in people with anxiety [89], stimulants and non-stimulants in people with ADHD [90], DBT in people with BPD [91], and mindfulness-based interventions [92,93]. In relation to HRV, it has been shown that HRV-biofeedback produces increases in HRV, and this is reflected in reduced ED and better ER at the behavioral level [79,80]. A major limitation of the current literature is that methodological differences across studies (e.g., type of intervention and duration, active vs. passive control arm, sample characteristics, type of imaging technique used) often lead to inconsistent patterns and findings that are rarely replicated in larger follow-up studies. Adopting transdiagnostic and computational approaches to the study of the effects of different interventions for ED on brain and body functioning, could help predict what intervention works best for people with specific clinical and demographic profiles.”

Comment 6: have you evaluated the inclusion of a table to summarize the evidence?

Response: We have not included a Table, but we have now summarized the main findings of our review on page 14:

“We narratively reviewed the evidence about the effectiveness of pharmacological and non-pharmacological interventions for reducing emotion dysregulation (ED) and improving emotion regulation (ER). Overall, based on the current evidence, the most used and effective are medications commonly used to treat depression (both SSRIs and NRIs), ADHD (both stimulants and non-stimulants), and psychotic symptoms (e.g., risperidone or aripiprazole); and psychological interventions or adaptations of such interventions (based on CBT, DBT, and mindfulness components), e.g., by integrating biofeedback components (HRV-biofeedback) or delivered via digital technologies (e.g., mobile or virtual reality).”

Comment 7: I agree that emotional dysregulation is a transdiagnostic elements, but I think that treatments reported in the review have been related to specific diagnoses. Have you evaluated if this aspect is balanced or not?

Response: This is a very interesting point, which we have now discussed as a limitation of the existing evidence, on page 15, as follows:

“While systematic reviews and umbrella reviews studying interventions for ED have been published (see, for example, [60]), these are often limited to one interventional domain (e.g., pharmacological or non-pharmacological), one type of intervention, and/or specific disorders (which collides with the theories about the transdiagnostic nature of ED). Further evidence synthesis studies, e.g., individual participant data network meta-analyses, are needed to elucidate the effects of different pharmacological and non-pharmacological interventions both on ED symptoms at behavioural level, and in relation to underlying bio-psycho-social factors.”

Comment 8: please include a paragraph about the limits of this revision. You should be aware of them, especially the possible biases and the possible exclusion of some papers.

Response: We have now added a brief description of limitations of our narrative review in the abstract and on page 15, as follows:

“We have identified some limitations of both our work and the reviewed literature, which shall be addressed in future research. This is a narrative review, and our search (although systematic) was only restricted to systematic reviews and meta-analyses, which could have led to selection bias, namely the selection of studies and the possible exclusion of others. While systematic reviews and umbrella reviews studying interventions for ED have been published (see, for example, [60]), these are often limited to one interventional domain (e.g., pharmacological or non-pharmacological), one type of intervention, and/or specific disorders (which collides with the theories about the transdiagnostic nature of ED). Further evidence synthesis studies, e.g., individual participant data network meta-analyses, are needed to elucidate the effects of different pharmacological and non-pharmacological interventions both on ED symptoms at behavioural level, and in relation to underlying bio-psycho-social factors. Lastly, most of the systematic reviews and/or meta-analyses included cross-sectional studies, which cannot provide conclusive information about the development of ER across development, and the factors affecting such developmental trajectories. For example, the fact that ER circuits continue to mature until young adulthood, highlights the importance of planning future studies aimed at understanding developmental trajectories of ER skills maturation, and explaining why differences in emotional processing and regulation between younger adolescents and older adults are often evident.”

Round 2

Reviewer 2 Report

Comments and Suggestions for Authors

The new version of the manuscript has been significantly improved. According to me, it is suitable for publication.